# RETention of urine After INguinal hernia Elective Repair (RETAINER study I and II)

**DOI:** 10.29337/ijsp.137

**Published:** 2021-04-23

**Authors:** Irish Surgical Research Collaborative, Stefanie M. Croghan, Christina A. Fleming, Helen M. Mohan, Deena Harji, Jarlath C. Bolger, Jessie A. Elliott, Michael Boland, Peter E. Lonergan, Patrick Dillon, David M. Quinlan, Des C. Winter

**Affiliations:** 1 Royal College of Surgeons, IE; 2 Irish Surgical Research Collaborative (ISRC), IE; 3 Department of Anaesthesia, University Hospital Limerick, IE

**Keywords:** Protocol, Inguinal hernia, Urinary retention, POUR, postoperative

## Abstract

**Purpose:**

Post-operative urinary retention (POUR) is a well-recognised complication of inguinal hernia repair (IHR). The magnitude of the problem is unclear, and contradictory evidence surrounds postulated risk factors. POUR risks patient distress, catheter-complications and a financial and logistical burden to services. Separately, in the field of IHR, there has been a lack of research into patients’ perceptions of surgical ‘success’. Our aim is to perform a two-phase, multi-centre prospective study to:

1. Assess the rate, risk factors and impact related to POUR post IH repair.
2. Develop and validate a patient reported outcome measure (PROM) for inguinal hernia repair.

**Methods:**

RETAINER I: We propose a 24-week prospective study with voluntary international participation in 4 week blocks. All patients undergoing elective IH repair (minimally-invasive/open) will be eligible. Standardised data collection will include patient and perioperative factors. Primary outcome will be development of POUR, defined as the need for insertion of a urinary catheter as determined by the treating clinician. Secondary outcomes will be identification of factors predisposing to POUR and the impact of POUR.

RETAINER II: A patient reported outcome measure will be developed using representative patient focus groups for item generation, from which an initial questionnaire will be developed and piloted. Validity, reliability, sensitivity and reproducibility will be assessed using the QQ-10 and standard psychometric methodology.

**Conclusions:**

Using an international multicentre collaborative approach will produce the necessary volume of patients, whilst capturing inter-centre variability, to accurately reflect POUR rates and allow analysis of risk factors. This patient pool will provide an excellent opportunity to develop a PROM using appropriate qualitative methodology.

**Highlights: RETAINER I & II Protocols:**

- RETAINER (RETention of urine After INguinal hernia Elective Repair) I is a prospective, multicentre, international observational study.
- RETAINER I aims to explore the *incidence of* and *risk factors for* urinary retention following elective inguinal hernia repair.
- Urinary retention following inguinal hernia repair has a marked impact on patients and creates a significant financial and logistical burden for hospital services.
- RETAINER II is a prospective, qualitative study, recruiting patients to guide the creation of a patient-reported outcome measure (PROM) for elective inguinal hernia repair.

## RETAINER I An Audit of Acute Urinary Retention Post Elective Inguinal Hernia Repair

### Introduction

Post-operative urinary retention (POUR) anecdotally occurs in a significant proportion of male patients following inguinal hernia repair. This has a potentially detrimental effect on both patients and services, due to the need for urinary catheterisation and admission.

The international literature reports an extremely wide range (0.4%–41.6%) of rates of acute urinary retention following inguinal hernia repair [1,2,3,4,5,6,7,8,9,10]. Potential peri-operative risk factors postulated from a mixture of prospective and retrospective studies are the choice of a laparoscopic approach [8,5], the use of spinal anaesthesia [11,12], bilateral inguinal hernia repair [1,2], increased duration of operative time [2], increased volume of perioperative fluids [13], use of specific anaesthetic agents [13], and increased use of narcotic analgesia [3]. Contradictory evidence surrounds many proposed risk factors, however, with some authors finding no statistically significant correlation of POUR with open versus laparoscopic approach [1], with choice of anaesthesia [1], with performance of synchronous bilateral IH repair [3], or with volume of intra-operative fluids infused [3]. Some studies have assessed patient related risk factors and identified increased BMI [2] and increased patient age [1,2] as being associated with higher rates of POUR. Again, some dispute exists, notably surrounding whether or not an existing diagnosis of benign prostatic hyperplasia (BPH) increases the risk of POUR [1,3].

Whilst urinary catheterisation may be considered a minor intervention, it is far from inconsequential. The need for insertion of a catheter can cause patients significant distress, prolong their hospital stay, restrict mobilisation [14] and risk complications. A recent prospective multi-centre trial assessing outcomes of short-term catheterisation of hospitalised patients (catheter indwelling for ≤3 days in 76%) found that one or more catheter-related complication were described by 57% (1184/2076) of patients at 30 day follow up [15]. Catheter associated UTIs (CAUTIs) are amongst the most common hospital acquired infections reported globally [16,17,18,19], have been described as the most common identifiable source of secondary blood-stream infection in the hospital setting [20], and are associated with the culture of rising numbers of resistant organisms [21]. Common non-infectious complications of short-term urinary catheters include blockage, leakage and haematuria [22]. More concerningly, accidental removal, urethral stricture or erosion (3.4%–16.7%) [22] and iatrogenic trauma during insertion (0.3–3%) [23,24,25] may occur, causing significant morbidity with potentially life-long consequences for the patient.

Furthermore, urinary retention following inguinal hernia repair has a significant impact on hospital services. Elective inguinal hernia repair is typically planned as a day-case procedure. The development of acute urinary retention in a patient mandates placement of a urinary catheter. This generally requires admission to hospital, with the alternative being catheter training with short-interval follow-up by specialist services. Undiagnosed or later evolving POUR may require Emergency Department attendance and/or admission in the early post-operative days. All scenarios pose a significant financial and logistical burden to hospital services. Post-operative urinary retention has been identified as the cause for unplanned admission in 20–25% of ambulatory general surgical procedures [26,27]. Whilst there is a lack of published data on the economic impact of this in the context of individual health systems, it inevitably distorts the internationally anticipated 36–65% cost saving expected with performing hernia repair as day-case surgery [28]. Should a complication of catheterisation occur, costs are greatly amplified; US evidence suggests that total costs incurred in the management of a catheter associated UTI exceed $1,000 [29] and estimates of €3,846–€9,064 per patient for the management of iatrogenic urethral trauma in Ireland have been declared [30,24].

### Rationale and hypothesis

Post-operative urinary retention following elective inguinal hernia repair may have a significant impact on patient morbidity and a high cost to healthcare services. Rates of POUR have not been formally evaluated in a global context, it is unclear what effect the surgical approach and choice of anaesthesia have on the incidence of POUR, and no risk stratification system is in place to preoperatively identify patients most likely to experience this complication.

Our hypothesis is that POUR is a significant complication of IH repair internationally, in both its incidence and its consequences. We propose that a prospective audit of this will assess the magnitude of the problem, and may identify patient and surgical factors increasing its incidence. Such findings would have potential to inform future research, which may explore ways to minimise and manage the complication of POUR in high-risk patient groups.

### Objectives

- To identify the rate of post-operative urinary retention (POUR) in male patients undergoing elective inguinal hernia (IH) repair in participating centres internationally.
- To assess preoperatively the International Prostate Symptom Score (IPSS) in this patient cohort, and where possible, to record a pre-operative post-void residual volume (PVR) in these patients, and to assess correlation of these variables with POUR.
- To record the surgical approach to hernia repair, the anaesthetic approach used, including record of all anaesthetic drugs used intraoperatively, and the post-operative analgesic regimen on the ward, to assess correlation of these variables with POUR.
- To examine the impact of POUR on patient morbidity and on hospital services (secondary aims).

### Design and Methods

- A multicentre, multinational prospective cohort study is proposed.
- Any hospital providing elective inguinal hernia repairwill be eligible to enrol patients. A named surgicalconsultant/attending will act as the local principalinvestigator/supervisor. Local data collection will bemanaged by teams that may incorporate students, surgical trainees/residents and consultants/attendingsat each participating centre. The study will be registered and approved by eachparticipating hospital’s audit committee or researchethics committee as appropriate.
- Patient eligibility
  - Patients 18 years or over undergoing elective inguinal hernia repair by any surgical approach are eligible for entry into this study.
  - Patients with a long-term indwelling catheter and those who routinely perform self-catheterisation to empty their bladder, those with any form of urinary diversion, those undergoing emergency operations and those unwilling or unable to consent to participation are to be excluded.
- Projected numbers
  - We aim to enrol a minimum of 40 centres in this study.
  - We propose a data collection period of 24 weeks, with a target enrolment period per centre of 12–16 weeks and a minimum enrolment period of 4 weeks. Flexibility in data collection is supported due to the ongoing COVID-19 pandemic.
  - The study is powered based on an incidenceof urinary retention of 5%. This would require minimum n = 600 participants to find a difference between main postulated risk factors. With a conservative estimate of 2 inguinal hernia repairs per site per week, we predict patient enrolment in comfortable excess of 600.
- Checks and balances
  - Upon enrolment, centres will submit their local hernia figures for the previous week.
  - Interim data analysis of primary outcome per centre will be performed at 8 weeks and extension of study period or recruitment of further centres arranged if required.
  - We acknowledge the potential impact of the global SARS-CoV-2 on target figures, and have identified a 24 week data collection period to allow extension or delayed starts in data collection periods in the instance of inadequate patient recruitment.
- Study phases
  - Pilot: A three-week pilot study has been undertaken in University Hospital Limerick. This has ensured feasibility and validated data collection techniques, with minor improvements made to the data collection form following same.
  - RETAINER I: The study will run over a minimum 4-week study period per centre, with a view to each centre participating in 1 - 5 × 4-week blocks from a designated 24-week period.
  - RETAINER II: Patients enrolled in phase 1 who agree to participate in phase 2 will be contacted once sufficient patients are enrolled to have an adequate sample for a focus group and a further sample for testing the questionnaire.

### Outcome Measures

#### Primary

The definition of the primary endpoint is the rate of post-operative urinary retention (POUR) following elective inguinal hernia in male patients, where POUR is defined as the need for insertion of a urinary catheter as determined by the treating clinician.

#### Secondary

- Pre-operative
  - Patient demographics.
  - Preoperative urological medications or urological diagnosis.
  - International Prostate Symptom Score (IPSS) of patient.
  - 1 or more post-void residual measurement (PVR) of patient where logistically feasible.
- Intra-operative
  - Surgical approach – laparoscopic versus open
  - Unilateral versus bilateral hernia repair
  - Anaesthetic approach: Local/Spinal/General
  - Anaesthetic agents used
  - Perioperative fluid volume infused
  - Duration of surgery
- Post-operative
  - Analgesia administered on ward
  - Time to voiding
  - Need for urinary catheter insertion
  - If catheter inserted, outcome – morbidity and service burden

### Data collection

Variables to be collected:

- Pre-operative
  - Patient age
  - Patient BMI
  - ASA grade
  - Preoperative medications for the treatment of bladder outlet obstruction or overactive bladder, or the use of alpha-blockers or medications with anticholinergic effect for other indications.
  - History of relevant urological diagnosis (benign prostatic hyperplasia/prostate cancer/urethral stricture/bladder neck stenosis/detrusor underactivity/detrusor overactivity) or of relevant urological procedure (radical prostatectomy/transurethral prostatectomy/bladder neck or urethral surgery/pelvic radiotherapy)
  - History of previous episode of acute urinary retention, and if so, whether spontaneous or provoked by C2H5OH, urinary tract infection (UTI) or constipation
  - Pre-operative International Prostate Symptom Score (IPSS) of patient
  - Pre-operative post-void residual measurement (PVR) of patient where logistically feasible
  - Day bowels last opened
- Intra-operative
  - Surgical approach – laparoscopic versus open
  - Unilateral versus bilateral hernia repair
  - Involvement of bladder in hernia
  - Anaesthetic approach: Local/Spinal/General anaesthesia
  - Anaesthetic agents used
    - Systemic: Glycopyrronium, diazepam, pentobarbital, propofol, isoflurane, methoxyflurane, halothane, muscle relaxants
    - Spinal: Anaesthetic and opioid use
    - Local: agent of choice, with/without adrenaline
  - Perioperative analgesics
  - Perioperative fluid volume infused
  - Duration of surgery
  - Intraoperative complications (recognised injury to bladder, ureter, nerves or bowel)
  - Mesh used and type
- Post-operative
  - Time to voiding
  - Need for urinary catheter insertion prior to discharge
  - Time of catheter insertion (hours post-operative)
  - Indication for catheter insertion as assessed by the treating clinician:
    - Failure to resume normal voiding on the day of surgery as clinically determined
    - Inability to void despite the urge to do so
    - Suprapubic pain deemed secondary to failure to void
    - A palpable bladder
    - An ultrasound bladder scan volume of >600ml
  - Presentation to ED/other healthcare venue with urinary retention within 72 hours of surgery
  - 30-day readmission
  - If urinary retention, outcome:
    - Method of decompressing the bladder (urethral catheter, self-intermittent catheter, suprapubic catheter)
    - Number of catheterisation attempts
    - Residual volume of urine
    - Digital rectal exam findings
    - Overnight admission
    - Timing of trial without catheter (TWOC)
    - Whether or not TWOC was successful
    - Alterations to medications (addition of alpha-blocker or 5-alpha reductase inhibitor; cessation of beta-3 agonist or anti-cholinergic)
    - Outcome if first TWOC unsuccessful
    - Complications: Acute kidney injury, urinary tract infection, accidental catheter removal with balloon inflated, pain/bladder spasm
    - Whether an inpatient urology consultation was sought
    - Whether inpatient urological intervention was required
    - Whether patient was discharged with a urinary catheter in-situ
    - Whether patient was taught self-intermittent catheterisation whilst an inpatient
  - Estimated cost analysis of the POUR (catheter and associated equipment, length of stay X cost of local hospital bed), cost of additional medications

### Data Collection Methods

- Data will be collected in a standardised database. It will be the responsibility of the local principal investigator (PI) to ensure that the data are password protected and kept on a secure local server. The database will be pseudonymised. However, a separate password-protected key document, including patient hospital identifier, will be kept by the local lead investigator for a period of 30 days to allow for outcome follow-up.
- Data collection points:
  - Each hospital site should identify all theatres where procedures are being performed. All consultant surgeons and anaesthetists involved in procedures should give prior consent for data collection.
  - Patient identification: patients should be identified in advance of admission from elective operating lists.
  - Pre-operative data: these should be collected from the patients’ medical records. Patients should be asked in clinic/at preoperative assessment or on admission to the day ward to complete the IPSS questionnaire. Where feasible, a post-void residual measurement (PVR) should be recorded with a bladder scanner.
  - Operative data: these should be recorded by the operating surgeon or one of the assistants participating in the operation.
  - Post-operative data: All patients should be followed for 30 days post-operatively. Local arrangements should be made but these include monitoring paper and electronic records, monitoring outpatient or ED attendance and reviewing results (e.g. catheter urine specimens) where relevant.
  - There should be regular local audit to ensure all eligible local patients are being enrolled and that data collection is as complete as possible.
- Validation of dataset:
  - The supervising consultant(s) will be required to submit the total number of elective inguinal hernia repairs performed in male adult patients at their institution during the selected study period, as reported by the institution’s coding department, to be able to identify the number of cases captured in the audit.
- Data collation:
  - Data will be submitted centrally via REDCAP hosted on Royal College of Surgeons of Ireland (RCSI) servers with all patient identifiers removed, and unique study ID used for each patient only. A local key, kept securely in a password protected document by the local lead investigator, will link patient identifier to study ID and be maintained for 30 days to allow input of short-term follow up data. This will then be destroyed, or, if the patient has consented to participation in RETAINER II, maintained until completion of this phase.

### Statistical analysis

- The statistical data from this study will be reported in accordance with the guidelines set by the STROBE (Strengthening of the Reporting of Observational Studies in Epidemiology) consensus statement [31]. Data will be analysed by RCSI statisticians. Data will be analysed in clinically relevant categories with Chi squared analysis used to detect differences between groups.
- All data will be anonymised prior to analysis. Binary logistic regression modelling will be used. Multivariable models will be built to produce odds ratios (OR) to account for the impact of predictive variables when assessing outcomes. The OR represents the odds of post-operative urinary retention occurring. Variable selection will be based upon those which are statistically significant at univariate analysis, and those which are significant clinically but not statistically.

## RETAINER II Patient Reported Outcome Measure for Inguinal Hernia Repair

### Introduction & Objectives

There is a significant need for better tools to measure what really matters to patients, increasingly recognised across surgical disciplines [32,33]. A lack of standardised tools to measure patient reported outcomes limits research in this area. There is currently no validated tool specifically to record patient reported outcomes in inguinal hernia repair. This is particularly relevant in the context of the current focus on mesh use in inguinal hernia repair. Recruiting patients participating in part 1 of the RETAINER study to also be involved in development of a PROM for inguinal hernia repair presents an opportunity to develop and validate a meaningful tool for future research.

### Design and Methods

This study is designed to develop and evaluate the psychometric properties of a disease-specific PROM instrument for patients undergoing inguinal hernia repair. The evaluation and development of a disease-specific instrument will be carried out in accordance with current FDA guidelines for developing PRO measures, to ensure high quality and standardisation of the development of our PROM in inguinal hernia surgery [34,35,36,37,38].

The research design for RETAINER II is based on recommended guidelines and will be developed in three phases: 1) item generation; 2) pre-testing and 3) RETAINER II PROM evaluation.

Phase 1, Item Generation, will be developed by undertaking a systematic review and narrative analysis of health-related quality of life (HrQoL) outcomes literature relevant to patients with an inguinal hernia. Focus groups will be conducted with patients with an inguinal hernia to understand important relevant outcomes to patients. The information combined from the systematic review and qualitative interviews will produce a provisional conceptual framework. This will be scrutinised by the project steering group prior to finalising the conceptual framework.

Phase 2 of this project will be the development and pre-testing of the provisional RETAINER II questionnaire. Items (questions) will have been generated from the qualitative work carried out in phase 1. The aim of this phase is the refinement of the provisional RETAINER II tool, with review of the tool to ensure clarity and to achieve consensus, thus leading to the development of the pre-test version of the instrument. This provisional tool will be administered to a select number of patients with an inguinal hernia. Using cognitive interview techniques and the face validity score QQ-10 [39], the instrument will be refined by clarifying any ambiguities in item wording, reviewing the relevance and appropriateness of the question stem and response options, as well as the instruments time-frame. Based upon the responses gained, the provisional RETAINER II will be revised to produce a preliminary version ready for field testing.

Phase 3, a field test will be undertaken to evaluate the RETAINER II tool for reliability and validity using psychometric techniques from Classical Test Theory [40], to ensure rigour and scientific credibility.

### Phase I: Item Generation

An initial systematic review and narrative analysis will be undertaken to identify HrQoL issues relevant to patients with inguinal hernia, this will be used to identify themes and concepts to help inform the interview guide. Supplemental information using content analysis from five focus group consisting of 3–4 patients each will be used to develop a conceptual framework.

#### Methodology

A traditional focus group will be used to identify issues important and relevant to patients with an inguinal hernia. The focus group will be guided by an interview guided generated from the systematic review to aid and stimulate discussion. Focus groups are an appropriate method for addressing a broad range of research questions across a variety of health-related domains. Focus group information is a reflection of the group dynamics and behaviour, with the advantage of memories and thoughts stimulated or prompted by comments made by other members of the group. The use of a focus group will enable data collection in an interactive manner.

#### Sampling Strategy

A purposive sampling strategy will be employed to ensure all relevant patient and clinical characteristics have been captured to ensure generalisability of the findings to a wider population.

#### Eligibility

##### Inclusion Criteria

- Patients undergoing elective inguinal hernia repair.
- aged ≥18 years
- ability to provide informed written consent to participate and
- ability to read and write in English or another language spoken fluently by a named collaborative investigator working as a focus group facilitator who is also capable of and willing to translate study documents into the language in question.

##### Exclusion Criteria

Patients will be excluded from the study in any of the following criteria apply. They:

- Have cognitive impairment
- Are unable to speak/read and/or write in English or one of the languages that the patient information leaflet and consent form have been translated into by native speakers within the wider study collaborative, with review by the relevant local ethics/audit committee/other overseer bodies according to local or national requirements
- Are unable to provide informed consent

#### Data Analysis

The focus group recordings will be transcribed verbatim, with clear indication of pauses, emphasised words, expressions of emotion and unintelligible speech. Conversational norms such as interruptions and overlapping speech will be preserved. All transcripts will be reviewed by the focus group facilitator for accuracy and clarification of ambiguities.

Using the principles of content analysis, transcript data obtained from the focus group will be analysed to identify themes and codes. A combined approach will be used, using inductive methods to identify new categories arising from the transcripts and deductive methods to build on the categories identified by current literature.

The information combined from the systematic review and focus group will produce a conceptual framework. This conceptual framework will be scrutinised and refined by the study steering group.

### Phase 2: Pre-Testing

The purpose of this phase is to reach consensus regarding the questionnaire format and structure to confirm that instructions and items are clear, understandable, relevant, and applicable. Face validity of the questionnaire will be assessed using the QQ-10 [39].

#### Eligibility

Patients fulfilling the eligibility criteria in for RETAINER II will be eligible to participate.

#### Methodology

A pre-test questionnaire will be developed based on the conceptual framework devised in Phase I. Items from existing question banks will be mapped to the conceptual framework to devise a questionnaire. The pre-test questionnaire will be tested with a sample of patients. It is estimated that approximately 20–30 patients will be needed to reach saturation, with no new issues arising. A postal questionnaire will be administered to patients to self-complete. The pack will contain the RETAINER II questionnaire and the QQ-10 questionnaire.

Quantitative assessment of face validity of the measure will be undertaken using the QQ-10. This is a validated measure to assess the face validity, feasibility and utility of instruments designed to assess HrQoL.

#### Data Collection

Data collected will relate to feedback on participants understanding of each question and associated response category and instructions, and to verbalise how patients produced answers, with emphasis on memory retrieval and subsequent judgements and decision making. The QQ-10 consists of 10 quantitative questions scored on a Likert scale and 3 qualitative questions covering the domains of value and burden of questionnaire completion.

#### Date Analysis

Review and analysis of information collected from cognitive interviews will be conducted as soon as possible after the interview, but as a minimum after every 3 interviews. This will enable any major flaws in the provisional questionnaire to be identified and revised before subsequent pre-testing with the revised version. This method of multiple rounds of pre-testing will determine whether the problem identified has been rectified and to ensure no new problems have been introduced.

The data from the QQ-10 questionnaire will be analysed both quantitatively and qualitatively. For quantitative analysis we used the QQ-10 scoring method. Likert ratings from strongly disagree to strongly agree (coded as 0–4) will be summed up separately for the first six questions comprising the value score (‘helped me communicate about my condition’, ‘relevant to my condition’, ‘easy to complete’, ‘included all the aspects of my condition I am concerned about, was enjoyable, would be happy to complete as part of routine care), and from the last four questions comprising the burden score (too long, embarrassing, complicated, upset me). Qualitative thematic analysis will be performed on comments received in response to the three free-text questions at the end of the QQ-10.

### Phase 3: Field Testing

A postal questionnaire pack will be administered to patients to self-complete. The pack will include the RETAINER II tool, as well as additional measures selected for validation purposes. A select group of patients who have completed the baseline questionnaire, will have a second (re-test) questionnaire pack after initial questionnaire completion.

#### Eligibility

Patients fulfilling the eligibility criteria in for RETAINER II will be eligible to participate.

#### Methods

An approximate sample of 50–100 patients will be purposively sampled ensuring representation of all subgroups. There is no formal sample size calculation for the development of PRO measures, however, recommended guidelines state 5–10 patients should recruited per item within the questionnaire [37]. We are aiming to develop a PRO measure with a maximum of 10 items, therefore our sample size is deemed to be sufficient. Consecutive patients will be identified and approached to participate. Qualitative analysis, using NVIVO 12 for Windows (QSR International Pty Ltd., Australia) will be performed independently (by NOSTRA and ISRC collaborative members D Harji and CA Fleming).

#### Test-Retest

A test-retest will be undertaken with a sub-sample of participants recruited for the final field test. Consenting participants will complete a second questionnaire pack 5–7 days after the first questionnaire pack (approximately 50 patients). The length of the test-retest interval must be short enough to ensure that clinical change is unlikely to occur, but sufficiently long to ensure that participants do not recall their responses from their first assessment. A short test retest interval is necessary to ensure stability is being evaluated, and not clinical change, which will underestimate reliability.

#### Recruitment and Consent Procedure

Local collaborators will identify eligible patients. A record of those identified as eligible, approached to participate, refusals, consenting patients and questionnaires returned will be made. The recruitment and consent methods described in the preliminary field test will be used. Additionally, the Patient Information Leaflet will allow participants an option of completing second questionnaire 5–10 days later. For patients who have agreed to a second questionnaire, a second questionnaire booklet will be sent to their home address with a self-addressed envelope. Stamped, self-addressed envelopes will be provided for the patients to return completed questionnaires to their local hospital.

#### Data collection/Assessments

Study data will be recorded by members of the clinical teams and by patients on questionnaire packs. Data will be returned to the research office at RCSI.

Assessments will be undertaken as follows:

- Registration and Baseline
- RETAINER II Questionnaire Booklet
- 7–10 day follow up questionnaire pack

#### Registration and Baseline Data

Baseline information will be recorded by:

- Patient initials and date of birth
- Gender
- Ethnicity
- Marital Status
- Education
- Centre code
- Name of clinical research staff member conducting registration
- Confirmation of eligibility
- Confirmation of written informed consent
- Unilateral/bilateral inguinal hernia
- Primary/recurrent hernia
- Open or laparoscopic hernia repair

#### RETAINER II questionnaire pack

The baseline questionnaire pack will include:

- The provisional RETAINER II questionnaire
- Additional questionnaires selected for validation purposes.
  - **HERQL**
    This is a validated measure assessing QoL in patients undergoing inguinal hernia repair with 14 questions related to pre-operative status and 6 questions related to post-operative status.
  - **Carolina Comfort Scale**
    This is a validated QoL measure used to assess common hernia related symptoms including pain, mesh sensation and limitation of movement during a number of activities [41].
  - **EuraHS-QoL**
    This is a validated measure assessing QoL in patients undergoing laparoscopic surgery for unilateral hernia using self-fixating mesh [42]. It consists of 3 broad domains and 9 questions.

#### Psychometric Analysis

Psychometric analysis will be undertaken using the principles of Classical Test Theory. Traditional psychometric methods evaluate rating scales in terms of data quality, acceptability, reliability and validity [43,44].

### Stages of Questionnaire Analysis

#### 1. Completeness of Data

Prior to assessing the proposed scale structure of the RETAINER II the completeness of the data must be analysed to identify missing data at the item and scale level, distribution of responses and floor/ceiling effects and descriptive analyses of item items. Data completeness concerns the extent to which items are completed by the target population and the total number of available data sets from which total scores are computable. The criteria for acceptable levels of missing data is <10%, for computable total scores >50% and for maximum endorsement frequencies is <80% (floor/ceiling effects <80%).

#### 2. Defining HRQoL Scales in RETAINER II

Multi-trait scaling will be used to determine whether the items of the RETAINER II will fit in with the proposed scale structure. The grouping of items into subscales and scales and the calculation of summated scores is based on four scaling assumptions:

1. Each item should have substantial correlation with the sum score computed from all the other items in that particular scale (item internal consistency).
2. Each item should have substantially higher correlation with the sum score of the rest of the items in the scale than with scales measuring other concepts (item discriminant validity).
3. For items in the same scale, the correlation between one item and the sum of the other items should be of similar magnitude for all items (equal item-to-total correlation).
4. Items in the same scale should have equal variance.

If the first and second criteria are fulfilled the current grouping of items is supported. If the third and fourth criteria are fulfilled the items can be summed without weighting; if not, weighting is normally suggested to achieve equal item-to-total correlations and equal variances [38,45,46,47]. Multitrait scaling will include analysing item frequencies, item and scale descriptive statistics including mean, standard deviation and variance, estimates of scale internal consistency, item intercorrelations and item-to-total correlations.

#### 3. Item Internal Consistency

Item internal consistency is determined by analysing the correlations between all items within a scale (item intercorrelations) and correlations between each item and the total scale (item-to-total correlations). Item intercorrelations indicate the extent to which the items are related. The recommended mean item-intercorrelation for a scale should exceed 0.30. However, items that are highly correlated may indicate redundancy, therefore it is recommended that item intercorrelations exceeding 0.70 should be removed. Item-to-total correlations indicate the strength of the relationship between individual items and the construct being measured and should exceed 0.3, this is also known as Item Convergent Validity.

#### 4. Item Discriminant Validity

Item discriminant validity gauges the extent to which items correlate more highly with the concept they are hypothesised to represent than with other concept. Item discriminant validity is supported and a scaling success declared when the item-to-total correlations for an item and its hypothesised scale is more than two standard errors higher than its correlations with another scale [38,45,46]. Item discriminant validity is also supported when item-scale correlations of <0.4 between an item and other scales in the questionnaire are observed.

#### 5. Equal Item-To-Total Correlation

Items can be summed without weighting to generate scores when they are substantially linearly related to the total score computed from all items in that group; measure at similar points on the scale, contribute equally to the total score variance and contribute equal proportions of information to the total score. These four criteria are satisfied when the items are internally consistent, have similar mean scores, variances and item-total correlations. However, when item-total correlations exceed the 0.30 criterion, the criteria of equivalent item means, variances and item-total correlations are considered satisfactory.

#### 6. Evaluation of Scale Reliability and Validity

Following revision and finalisation of the scale structure of the LRRC-QoL tests of reliability and validity of the scales will be undertaken.

##### 6 a. Reliability

Reliability is the degree to which the questionnaire is free from random error. This is measured by the internal consistency of a scale and reproducibility of the questionnaire using the test-retest measure. Internal consistency measures the homogeneity of a scale ensuring all items are sufficiently similar, thus ensuring scale reliability using alpha coefficients (Cronbach’s Alpha). Cronbach’s Alpha indicates the extent to which items in a scale are interrelated by comparing the variance of the total score to the sum of the variances of the individual items. As the correlations between the items increase, the variance for the total score increases and alpha coefficients approximate unity. Internal consistency is considered to be good when Cronbach’s alpha co-efficient exceeds 0.70.

##### 6 b. Test-Retest

The test-retest measure assesses the stability of the questionnaire over a period of time during which the patients clinical state remains stable. The Intraclass coefficient (ICC) is used to measure the strength of agreement between repeated measures, by assessing the proportion of total variance of an observation that is associated with the between patient variability. If the ICC is large this suggests the random variability is low and a high proportion of the variance is attributable to the variation between patients. The measurements are thus described to have high reliability. Conversely, if the ICC is low, the random error variability dominates and the measurements have low reliability. A ICC score of 0.7 is recommended to ensure good test-retest reliability. The ICC will be calculated using a fixed-effects analysis of variance (ANOVA) model.

##### 6 c. Validity

###### Convergent Validity and Divergent Validity

Convergent validity reflects the correlation between individual assessment tools measuring the same phenomenon. Pearson’s product moment correlation will be used to analyse correlation between items and scales of RETAINER II, and HERQL, CCS and EuraHS-QoL questionnaires. Pearson’s values of greater than 0.4 are considered to be highly correlated. It is hypothesised that the scales in the RETAINER II questionnaire will not correlate with the HERQL, CCS and EuraHS-QoL unless they address similar themes.

#### 7. Known-Groups Comparison

The method of known-groups comparison will be used to assess the ability of the RETAINER II to distinguish between patients differing in clinical status. The clinical parameter hypothesised to form mutually exclusive patients for subgroup comparison included hernia type (primary and recurrent), hernia location (unilateral and bilateral) and surgical approach (open or laparoscopic). The independent student t-test will be used to examine differences in mean scores in 2 groups and the one-way analysis of variance (ANOVA) for more than 2 groups.

## Authorship for RETAINER I and II

- A collaborative authorship model, using The National Research Collaborative & Association of Surgeons in Training Collaborative Consensus Group Guidelines will be used, as demonstrated in a previous publication by our collaborative [48].
- Preparation of a manuscript for publication will be performed by a writing committee.
- Collaborators contributing to the running of the study will be listed as ‘PubMed’ citable authors as part of the RETAINER study group.
- There will be a lead trainee for each site, who will be responsible for submitting the names of all authors from that site to the collaborative.
- There is no minimum number of data collectors eligible for authorship from each site – any person who has made a significant contribution as confirmed by the local site lead and consultant PI may be included.
- There should be at least one consultant/attending surgeon who agrees to act as a PI for each site.
- It is the local site lead and PI’s responsibility to ensure validity of the submitted dataset.

## Definitions

The following definitions will be used for this study:

- Post-Operative Urinary Retention: In the absence of a universally accepted definition [13] POUR will be defined in this study as the need for insertion of a urinary catheter as determined by the treating clinician
- American Society of Anaesthesiologists (ASA) physical status classification system is a system for assessing the fitness of patients before surgery. These are:
  1. A normal healthy patient
  2. A patient with mild systemic disease
  3. A patient with severe systemic disease
  4. A patient with severe systemic disease that is a constant threat to life
  5. A moribund patient who is not expected to survive without the operation
- Method of operation:
  - Laparoscopic
    - Transabdominal preperitoneal (TAPP) repair
    - Totally extraperitoneal (TEP) repair
  - Laparoscopic converted to open: procedure attempted laparoscopically with necessitation of conversion to an open procedure.
  - Open procedure: performed as a planned open procedure
- Duration of procedure:
  Time from first skin incision to final skin closure.
- 30-day post-catheterisation complications – complications occurring within 30 days from the date of catheter insertion including:
  - (CAUTI): A UTI in a patient who had an indwelling urinary catheter in place at the time of, or within 48 hours prior to infection onset.
  - Urinary tract infection (1) A culture of pure organisms >100,000 cfu/ml from a catheter specimen of urine in a symptomatic patient (elevated inflammatory markers or pyrexia in the absence of a more likely source, suprapubic pain, frequency/dysuria (if catheter removed). (2) Urine dipstick positive for nitrites along with leukocytes +/– blood/protein in a symptomatic patient (ideally this should be correlated with a positive urine culture).
    - Traumatic catheterisation (2 or more failed attempts at catheterisation, clearly visible haematuria or clots immediately post catheterisation, inflation of balloon in urethra, urethral injury diagnosed by a urologist)
- Unplanned Admission – need for overnight admission of a patient planned for ambulatory (day-case) surgery.
- Re-attendance due POUR: attendance at ED/Surgical Assessment Unit/Day Ward within 72 hours of surgery due to inability to void requiring insertion of urinary catheter, whether or not admitted.
- Readmission is defined as any admission following discharge necessitating an overnight stay.
- Length of stay: calculated from the date of admission to the date of discharge.
